# Identifying psychological distress in dementia caregivers: unmet needs and the urgent call for support

**DOI:** 10.1002/alz70858_106330

**Published:** 2025-12-26

**Authors:** Cristina Festari, Claudio Singh Solorzano, Giuliano Binetti, Roberta Rossi, Michela Pievani, Gianbattista Tura, Barbara Borroni, Orazio Zanetti

**Affiliations:** ^1^ IRCCS Istituto Centro San Giovanni Di Dio Fatebenefratelli, Brescia, Italy; ^2^ MAC‐Memory Clinic and Molecular Markers Laboratory, IRCCS Istituto Centro San Giovanni di Dio Fatebenefratelli, Brescia, Italy; ^3^ IRCCS Fatebenefratelli, Brescia, Italy; ^4^ IRCCS Centro San Giovanni di Dio ‐ Fatebenefratelli, Brescia, BS, Italy; ^5^ University of Brescia, Brescia, Italy; ^6^ IRCCS Centro San Giovanni di Dio Fatebenefratelli, Brescia, Italy

## Abstract

**Background:**

Informal caregivers of individuals with dementia are particularly vulnerable to psychological distress, including anxiety, depression, and caregiver burden. In recent years, healthcare professionals have intensified efforts to support this population. This cohort study aims to identify psychological profiles of caregivers and evaluate the effectiveness of the Italian healthcare system in addressing their needs.

**Method:**

A total of 607 informal caregivers of outpatients of the Memory Clinic of Brescia (Italy) completed an anonymous questionnaire. The survey collected sociodemographic characteristics, distress symptoms (i.e., burden, assessed with the ZBI, and anxiety, depression, measured using PHQ‐4) and perceived care needs. An ad‐hoc scale, listing available supportive services, enabled classification into three groups: “Supported” (caregivers already receiving assistance), “Requiring” (caregivers in need of support), and “Unengaged” (caregivers not perceiving a need for care). Latent Profile Analysis (LPA) was conducted on 534 complete questionnaires to identify distinct distress profiles based on ZBI and PHQ‐4. Chi‐squared tests and *p*‐values were used to assess significant differences in frequencies and means.

**Result:**

The majority of respondents were women (75%), aged 50–69 (56%), and employed (59%). Overall, caregivers reported mild‐to‐moderate burden (mean±SD:36.82±18.43), no depression nor anxiety (2.03±1.82; 2.85±1.90, respectively). LPA identified three distress profiles: (A) resilient caregivers (*n* = 185; 34.7%), with minimal burden (20.13±11.12), anxiety (1.04±0.89), and depression (0.48±0.68), (B) At‐risk caregivers (*n* = 223; 41.8%) with moderate burden (39.56±12.11), anxiety (2.90±1.19), and depression (1.90±1.01); (C) a distressed caregivers (*n* = 127; 23.8%) experiencing severe burden (*M* = 56.57), anxiety (*M* = 5.34), and depression (*M* = 4.70). The Distressed group was more likely to cohabit (*p* < .05) with a more oppositional patient (*p* < .001), provide extensive practical assistance (*p* < .05) and dedicate more caregiving hours (*p* <001). A correlation analysis of the profiles and the needs survey reveals that 61% of caregivers are requiring support, with this need being significantly more prevalent among at‐risk caregivers (*p* < .05).

**Conclusion:**

The findings highlight the substantial emotional distress experienced by dementia caregivers, particularly those living with highly dependent and oppositional patients. A significant proportion of caregivers remain unsupported. Strengthening healthcare services for caregivers is essential to improving both their quality of life and the sustainability of dementia care.

